# Pan‐cancer analyses reveal multi‐omic signatures and clinical implementations of the forkhead‐box gene family

**DOI:** 10.1002/cam4.6312

**Published:** 2023-07-04

**Authors:** Xiaoman Bi, Dehua Zheng, Jiale Cai, Dahua Xu, Liyang Chen, Zhizhou Xu, Meng Cao, Peihu Li, Yutong Shen, Hong Wang, Wuping Zheng, Deng Wu, Shaojiang Zheng, Kongning Li

**Affiliations:** ^1^ Cancer Institute of The First Affiliated Hospital College of Biomedical Information and Engineering Key Laboratory of Tropical Translational Medicine of Ministry of Education Hainan Medical University Haikou China; ^2^ Department of Breast Thoracic Tumor The Second Affiliated Hospital of Hainan Medical University Haikou China; ^3^ School of Life Sciences, Faculty of Science The Chinese University of Hong Kong Hong Kong China; ^4^ Key Laboratory of Emergency and Trauma of Ministry of Education, Key Laboratory of Tropical Cardiovascular Diseases Research of Hainan Province, Hainan Women and Children's Medical Center Hainan Medical University Haikou China

**Keywords:** database, Forkhead box, multi‐omics, pan‐cancer, transcription factor

## Abstract

**Background:**

Forkhead box (FOX) proteins belong to one of the largest transcription factor families and play crucial roles in the initiation and progression of cancer. Prior research has linked several FOX genes, such as FOXA1 and FOXM1, to the crucial process of carcinogenesis. However, the overall picture of FOX gene family across human cancers is far from clear.

**Methods:**

To investigate the broad molecular signatures of the FOX gene family, we conducted study on multi‐omics data (including genomics, epigenomics and transcriptomics) from over 11,000 patients with 33 different types of human cancers.

**Results:**

Pan‐cancer analysis reveals that FOX gene mutations were found in 17.4% of tumor patients with a substantial cancer type‐dependent pattern. Additionally, high expression heterogeneity of FOX genes across cancer types was discovered, which can be partially attributed to the genomic or epigenomic alteration. Co‐expression network analysis reveals that FOX genes may exert functions by regulating the expression of both their own and target genes. For a clinical standpoint, we provided 103 FOX gene‐drug target‐drug predictions and found FOX gene expression have potential survival predictive value. All of the results have been included in the FOX2Cancer database, which is freely accessible at http://hainmu‐biobigdata.com/FOX2Cancer.

**Conclusion:**

Our findings may provide a better understanding of roles FOX genes played in the development of tumors, and help to offer new avenues for uncovering tumorigenesis and unprecedented therapeutic targets.

## 1 INTRODUCTION

The forkhead box (FOX) transcription factor family is a group of evolutionarily conserved proteins that play crucial roles in the initiation and progression of cancer.^1^, ^2^ To date, approximately 50 FOX genes have been found in humans, which have been classified into 19 subfamilies (FOXA to FOXS) based on the sequence homology of their evolutionarily conserved forkhead DNA‐binding domains.^3^, ^4^ In addition to the conventional function of controlling gene expression as transcription factors, the poorly conserved domains across the family endow FOX proteins with a variety of diverse roles. Consequently, the deregulation of FOX factors has demonstrated a multifaceted involvement in the inception, maintenance and progression of malignancies.^5^, ^6^, ^7^, ^8^, ^9^, ^10^

FOX family members exhibit substantial tissue and tumor heterogeneity. Pan‐tissue analysis revealed that different FOX genes can either express ubiquitously or in a tissue‐specific manner.^1^, ^11^ Particularly, FOX genes within the same subfamily might have distinct expression pattern; for instance, FOXO1 and FOXO3a are almost ubiquitously expressed while FOXO6 preferentially expressed in nerve tissue.^12^ Likewise, an increasing number of studies have revealed the inter‐tumor heterogeneity of FOX genes; in other words, FOX genes have a double‐edged effect on cancers as both oncogenes and tumor suppressor genes.^13^ FOXM1 and its regulatory network have been demonstrated to be the major predictor of adverse outcomes in 39 human malignancies, whereas most FOXO factors are regulators of cellular homeostasis and putative tumor suppressors.^14^, ^15^ Moreover, such duality has also been observed in the same FOX gene, where overexpression of FOXA1 is associated with a favorable prognosis for breast cancer while indicates a poor clinical outcome for prostate cancer.^16^, ^17^ Together, these studies demonstrate the complexity and interconnectedness of FOX genes involved in cancer progression. However, only a few FOX genes have been linked to the crucial process of carcinogenesis, our understanding of how FOX factors function and co‐regulate in various human malignancies is still limited.

To date, a limited number of studies have been conducted to investigate the transcriptomic/epigenomic alterations of FOX genes from a multi‐cancer perspective.^18^, ^19^, ^20^ However, the overall picture of the FOX gene family across human cancers is largely elusive due to the studies mainly focused on a single FOX gene or particular molecular data types. Taking advantages of multi‐omics profiling and integration, we conducted a thorough investigation of the broad molecular signatures of the FOX gene family across 33 human cancers, aim to reveal molecular etiology and novel putative therapeutic targets.

## 2 MATERIALS AND METHODS

### 2.1 Collection of FOX genes

We selected terms like “FOX gene family” and “Forkhead Box” based on literature mining technologies for cyclic searches in the PubMed database, merging them with the DAVID database^21^ to screen 49 FOX genes with official gene symbol and Entrez ID (Table S1).

### 2.2 Somatic mutation and copy number alteration analysis

Somatic mutation data and gene‐level CNV data were downloaded from UCSC Xena^22^ and the dataset of MSS mixed solid tumors.^23^ The mutation frequency of FOX gene is the proportion of the sample with mutations in a specific cancer. The CNV amplification and deletion frequency of FOX gene was calculated as the proportion of samples with CNV amplification or deletion in a specific cancer. The Pearson correlation between CNV value and mRNA expression of each gene was calculated using the cor.test function in the R programme.

### 2.3 Differential expression analysis of FOX genes

Gene expression profiles were downloaded from UCSC Xena and merged microarray‐acquired data sets (MMDs, Affymetrix Human Genome U133 Plus 2.0).^24^ Using the limma package in the R programme, differential expression analysis for cancer types with ≥10 matched normal samples was carried out. Differentially expressed genes were determined to have log2 (fold‐changes) >1 and *p*_adjusted_ < 0.05. The expression profiles were all log2 transformed.

### 2.4 DNA methylation analysis of FOX genes

DNA methylation 450K data were downloaded from the UCSC Xena database. Individual genes were assigned to Illumina methylation array probes. The probes that corresponded to promoter regions were retained. Beta‐means were computed for FOX gene samples with multiple CPG sites, while FOX genes with a single CPG site were retained. The Wilcoxon rank sum test and deltaR values, which measure methylation up‐ and downregulation alterations, were used to compare the levels of methylation in normal and cancer samples. Genes with *p* < 0.05 were identified as differentially methylated genes. The cor.test function in the R program was used to determine the Pearson correlation between DNA methylation beta values and mRNA expression of each gene.

### 2.5 Identification of dysregulated FOX interactions

For 16 malignancies (matched normal samples ≥10), the Pearson correlation coefficients (PCC) for FOX genes were calculated in cancer samples and normal samples and ranked in absolute value. The correlation coefficients of 10,000 randomly chosen gene pairings for each gene encoding a protein were then calculated in cancer samples and normal samples, and the results were ordered by absolute values.

For each FOX‐FOX gene pair, the number of 10,000 coding protein gene pairs with correlation coefficients greater than FOX‐FOX gene pair was counted, and the resulting number was divided by 10,000 to determine the new *p*‐value for each FOX‐FOX gene pair. Both the normal co‐expression networks and the cancer co‐expression networks were built using FOX–FOX gene pairings with *p*‐values under 0.01.

### 2.6 Identification of dysregulated FOX–target interactions

Target gene data for FOX genes were obtained from the ChIP‐Atlas (http://chip‐atlas.org),^25^ and target genes with significance scores greater than 100 were selected for analysis. To identify dysregulated FOX‐target interactions, the altered R was computed for the expression of the FOX‐target pair in the 16 cancer samples compared with normal samples in order. The estimated altered R was as follows:

$$\mathrm{\Delta R}={\mathrm{Cor}}_{\text{cancer}}\left(A,B\right)-{\mathrm{Cor}}_{\text{normal}}\left(A,B\right)$$

Cor_cancer_ (A, B) is the R of FOX‐target pair in cancer samples, and Cor_normal_ (A, B) is the R of a FOX‐target pairs in normal samples.^26^ Based on the value of ΔR, dysregulated FOX‐target interactions were classified as gain patterns (ΔR > 0) and loss patterns (ΔR<0).

The cancer/normal labels were permuted 1000 times at random on the presumption that the ΔR of a certain FOX‐target pair is significant. The significant *P*‐value for each R was as follows:

$$P=\frac{\sum_{i=1}^{N}S_{i}}{N}$$

For a certain FOX‐target pair, S_i_ =1 denotes that the value of random R was greater than the real one; otherwise, S_i_ = 0.

### 2.7 Enrichment analysis

Metascape^27^ was used for performing enrichment analysis of the gain (ΔR > 0) and loss (ΔR < 0) target genes. The pathways shared by more than three cancers were selected for presentation.

### 2.8 Survival analysis

Clinical data of patients with 33 cancer types were obtained from the UCSC xena database and E‐MTAB‐1980 cohort (*n* = 101, clear cell renal cell carcinoma).^28^ Cancer patients were divided into different groups based on the median value of FOX gene expression, and Cox regression was used to evaluate the association between FOX gene expression and survival. Kaplan–Meier survival curves for overall survival (OS) were also plotted. The differences in OS between different groups were examined using the log‐rank test, and the results were visualized by Kaplan–Meier survival curve.

### 2.9 Drug activity analysis

The IC_50_ data of drugs and gene expression data for all cell lines were obtained from the GDSC database.^29^ For each FOX‐drug pair, PCC between FOX gene expression and IC_50_ were calculated, and pairs with |PCC| > 0.2 and *p* < 0.05 were retained. Drug targets and related pathways were also retrieved from GDSC. PCCs for the FOX genes and drug‐target genes were calculated, and pairs with |PCC| > 0.2 and *p* < 0.05 were retained. The Sankey plot was used to display pairs with |PCC| > 0.2.

## 3 RESULTS

### 3.1 Global genomic alteration of FOX genes across 33 human cancers

**FIGURE 1:**
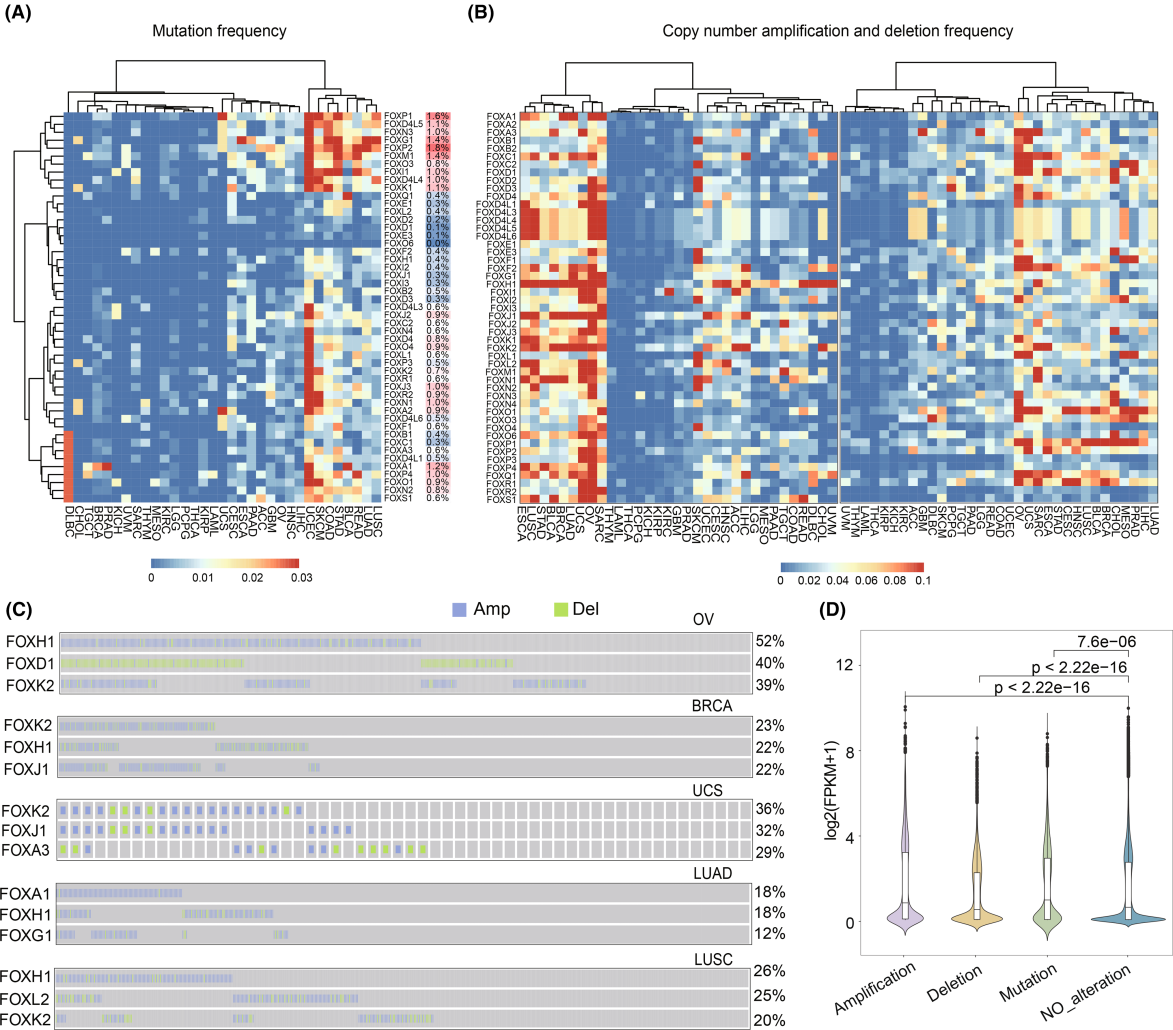
Genomic landscape of cancer‐related FOX gene alterations. Heatmaps showing the frequency of somatic mutations (A) and copy number variation (B) in the FOX genes across 33 human cancers, respectively. (C) The top three FOX genes' copy number variation patterns in five cancers they are associated with were displayed. Each row represents a gene, and each column represents the copy number variation status of a patient. Blue and green represents amplification and deletion, respectively. The right labels show the alteration rates of each gene. (D) The effect of genomic alterations of the FOX gene on their gene expression is depicted using a violin plot. *P*‐value (Wilcoxon test) are shown in the figure.

To gain insight into the genomic features of the FOX gene family in cancers, we first calculated the mutation frequency in the pan‐cancer cohort of 10,004 patients across 33 human cancers. Genomic analysis revealed that somatic mutations in FOX genes can be detected in 17.4% of tumor patients, with FOXP2 having the highest mutation rate (Figure 1A). This finding was further validated by a solid tumor cohort (Figure S1). Despite the difference in mutation rate, which can be partially attributed to the limited number of cancer types and samples, the results verified the robustness of genomic findings. Approximately 50% (10 out of 19) of FOX subfamilies shared similar mutation frequencies among their members, but the remaining subfamilies displayed heterogeneity. It should be noted that several FOX genes were highly mutated in certain cancer types despite that their overall mutation rates were rather modest, such as FOXQ1, which was particularly extensively mutated in BLCA. Overall, the FOX gene family exhibited a strong cancer type‐dependent genomic alteration pattern. Cancer types with the highest mutation frequency were observed in several cancer types including two types of lung cancers (LUSC and LUAD), two types of colon cancers (READ and COAD), BLCA, STAD, SKCM, and UCEC.

In addition to analysis of point mutations, we investigated copy number variation (CNV) in FOX genes. Several FOX subfamilies (FOXH, FOXJ, FOXK, FOXQ, and FOXS) showed extensive amplification in copy numbers. The FOX genes were predominately occurred in SARC, LUAD, LUSC, BLCA, STAD, ESCA, and three types of Pan‐Gyn cancers (BRCA, OV, and UCS). (Figure 1B,C). It suggests that highly‐amplification of FOX genes may account for the cell proliferation of these cancer types. In contrast to amplification, copy number loss showed a less prominent pattern of FOX genes.

To confirm the impact of genomic alterations on gene expression, we investigated the correlation between genomic alterations and their transcriptomic changes. As expected, patients with amplification of FOX genes had higher expression compared to those without alterations (*p* < 2.22e‐16), while either the deletion (*p* < 2.22e‐16) or point mutation (*p* = 7.6e‐6) account for a lower expression (Figure 1D). These results suggested that the genomic alterations of FOX genes may have a significant impact on their transcriptomic changes.

### 3.2 Heterogeneous expression of FOX genes across cancer types

**FIGURE 2:**
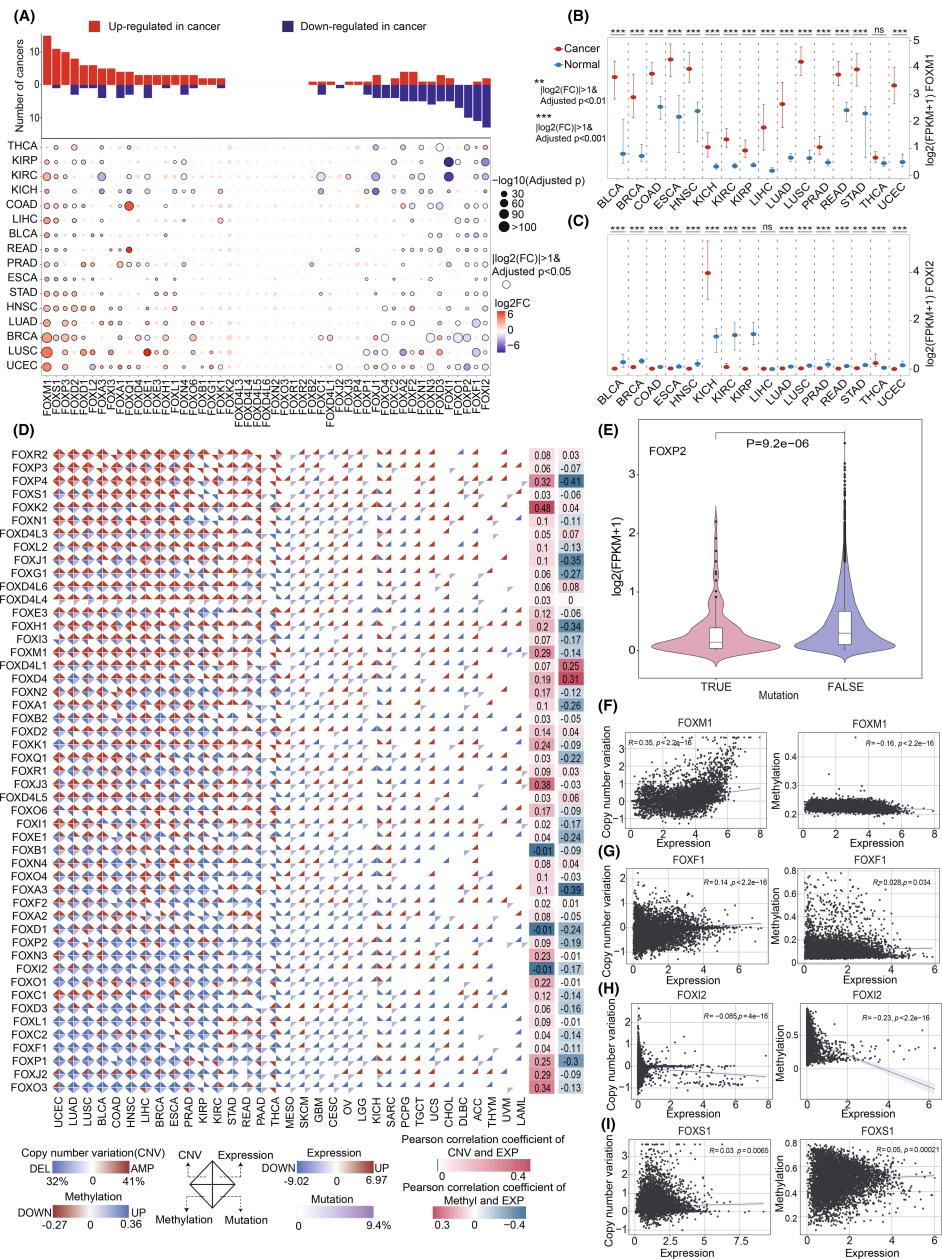
FOX gene transcriptome alteration of and its correlation with genomic instability in cancer. (A) Bar plots showing the number of cancer types in which each FOX gene exhibited upregulation or downregulation. Bubble plots showing the FOX gene expression between cancer and normal samples. The expression of FOXM1 (B) and FOXI2 (C) in cancer and normal samples across cancer types was shown using box plots. The middle line represents the median, with the upper and lower quartiles denoting the box's boundaries. ns, not significant. (D) Multi‐omics analysis of the FOX gene family. Each diamond is made of four pieces. The upper left of the diagram shows the copy number variation of the FOX gene, the upper right shows the gene expression level, the lower left shows the methylation level, and the lower right shows the mutation frequency. The right labels show the relative *R*‐values for expression and CNV, as well as for expression and methylation level. (E) The association between FOXP2 mutation and its gene expression. Correlation plots showing the correlation between gene expression and copy number variation or methylation in the FOXM1 (F), FOXF1 (G), FOXI2 (H) and FOXS1 (I) genes. CNV, copy number variation. *R*, Pearson correlation coefficient. *p*, p‐value of Pearson correlation.

Despite the fact that dysregulated expression of certain FOX genes has been observed in a few cancers,^30^, ^31^ the expression pattern of the entire FOX gene family across cancer types remains unclear. Filtering for cancer types with at least 10 matched tumor‐normal samples, 16 cancer types were selected for further analysis. Overall, FOX genes displayed high expression heterogeneity across these cancer types (Figure 2A). Forty out of forty‐nine (81.6%) FOX genes were differentially expressed in at least one cancer type. It is noteworthy that FOXM1 showed elevated expression in all 16 cancer types, including 15 cancers with statistical significance and 1 cancer (THCA) without significance (Figure 2B). By contrast, FOXI2 was downregulated in 13 of 16 malignancies but showed conspicuous upregulation in KICH (Figure 2C). Additionally, we also identified several genes, including FOXS1, FOXP3, and FOXD2, showed elevated expression in a variety of tumors, while FOXF1, FOXP2, and FOXO1 were downregulated in the majority of cancers. These significant findings were supported by independent microarray‐derived datasets (Figure S2).

To systematically depict the changes of multi‐omics in each FOX gene across cancers, we investigated the correlation between copy number alteration, methylation level, point mutation, and transcriptome changes (Figure 2D). Overall, the methylation patterns of the FOX genes' promoter regions revealed a negative association with gene expression, while copy number alteration displayed a positive correlation (Figures S3 and S4). We next investigated whether the low expression of FOXP2, which is the most often mutated FOX gene in cancers, was due to its high mutation burden. As expected, FOXP2 mRNA levels were significantly lower in FOXP2 mutation carriers than in noncarriers (Wilcoxon test, *p* = 9.2e‐6) (Figure 2E). In addition, we found a positive correlation between the expression of FOXM1 and its copy number alteration (*R* = 0.35, *p* < 2.2e‐16), and a negative correlation with its degree of methylation (*R* = −0.16, *p* < 2.2e‐16) (Figure 2F). Likewise, the expression of FOXF1 or FOXI2 genes and either their CNV or methylation level also showed high associations (Figure 2G,H). While neither FOXS1's CNV nor methylation contributed to its dysregulated expression, thus suggesting that there may be additional contributing factors (Figure 2I).

### 3.3 Disruption of the FOX gene co‐expression network in cancer

**FIGURE 3:**
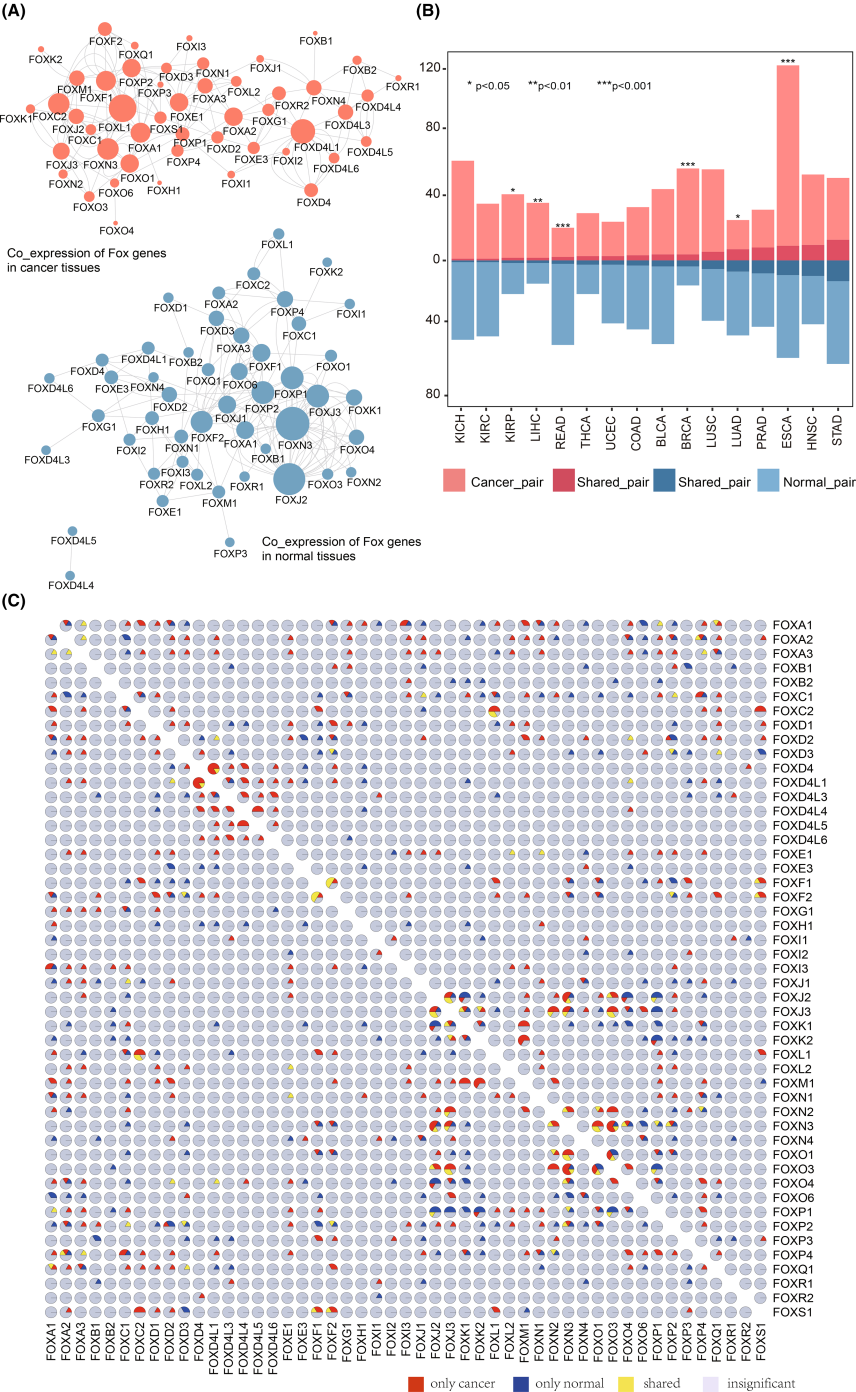
Global disruption of FOX gene co‐expression network in cancer. (A) FOX gene co‐expression network in cancer and matched normal samples. (B) The number of FOX co‐expression pairs in cancer and normal tissues is displayed in a bar plot for each cancer type. Darker color signified the co‐expression pairs shared by both cancer and normal samples. (C) FOX gene co‐expression network in normal and tumor tissues. Each pie chart shows the percentage of co‐expression pairs that present in cancer samples only (red), present in normal samples only (blue), shared by both cancer and normal samples (yellow), and insignificant ones (gray).

Multiple FOX genes shared a similar transcriptomic pattern (Figure 2A), which is consistent with the notion that genes in a family are expected to be co‐regulated and expressed to exert their functions.^32^, ^33^, ^34^ Therefore, we constructed the co‐expression networks of FOX genes using Pearson correlation coefficient to characterize the consensus co‐expression (Figure S5). A total of 746 and 672 co‐expression relationships were detected successfully across 16 tumor tissues and their matched normal tissues, respectively (Tables S2 and S3). Figure 3A depict that the co‐expression network generated from tumor tissues had FOXL1 and FOXD4L1 in the core, whereas the network from normal tissues had FOXN3 and FOXJ2 in the center. Our results demonstrated that the FOX co‐expression relationships are globally disrupted in cancer tissues, despite the fact that there was no markedly difference in the number of co‐expressions. Detailed information of tumor‐specific and normal‐specific co‐expressions is provided in Table S4 and S5.

To determine which type of cancer is responsible for the perceptible alterations, we examined changes in the FOX co‐expression gene pairs for each cancer type in two networks. Four cancer types (KIRP, LIHC, BRCA, and ESCA) showed a significant increase in the number of co‐expression pairs, while two types (LUAD and READ) exhibited a decrease when compared to normal tissues (Binomial test, *p* < 0.05, Figure 3B). The conservative relationship for sustaining the basic biological functions necessary for human health may be represented by the FOX co‐expression gene pairs that are shared by normal tissues and tumor tissues. We next investigated the changes of FOX co‐expression in six cancers with substantial changes and their matched normal tissues. FOXD4 and FOXD4‐Like genes (FOXD4L1, etc.) were significantly co‐expressed in 6 cancers, mostly in breast cancer, but not in their corresponding normal tissues (Figure 3C). It is noteworthy that both the CNV patterns and the gene expression patterns of the FOXD4‐Like genes exhibited consistency as well (Figure 1B and Figure 2A). Twenty FOX genes from seven FOX subfamilies (FOXJ to FOXP) correlated with each other either in normal tissues or matched cancer types. Taken together, it is possible that the FOX genes' redundant co‐expression may serve as a guarantee against accidental loss of function, and targeting these tumor‐specific co‐expressions may lead to new insights into the role of FOX genes in cancer.

### 3.4 Regulatory patterns of FOX genes on the target gene in cancer

**FIGURE 4:**
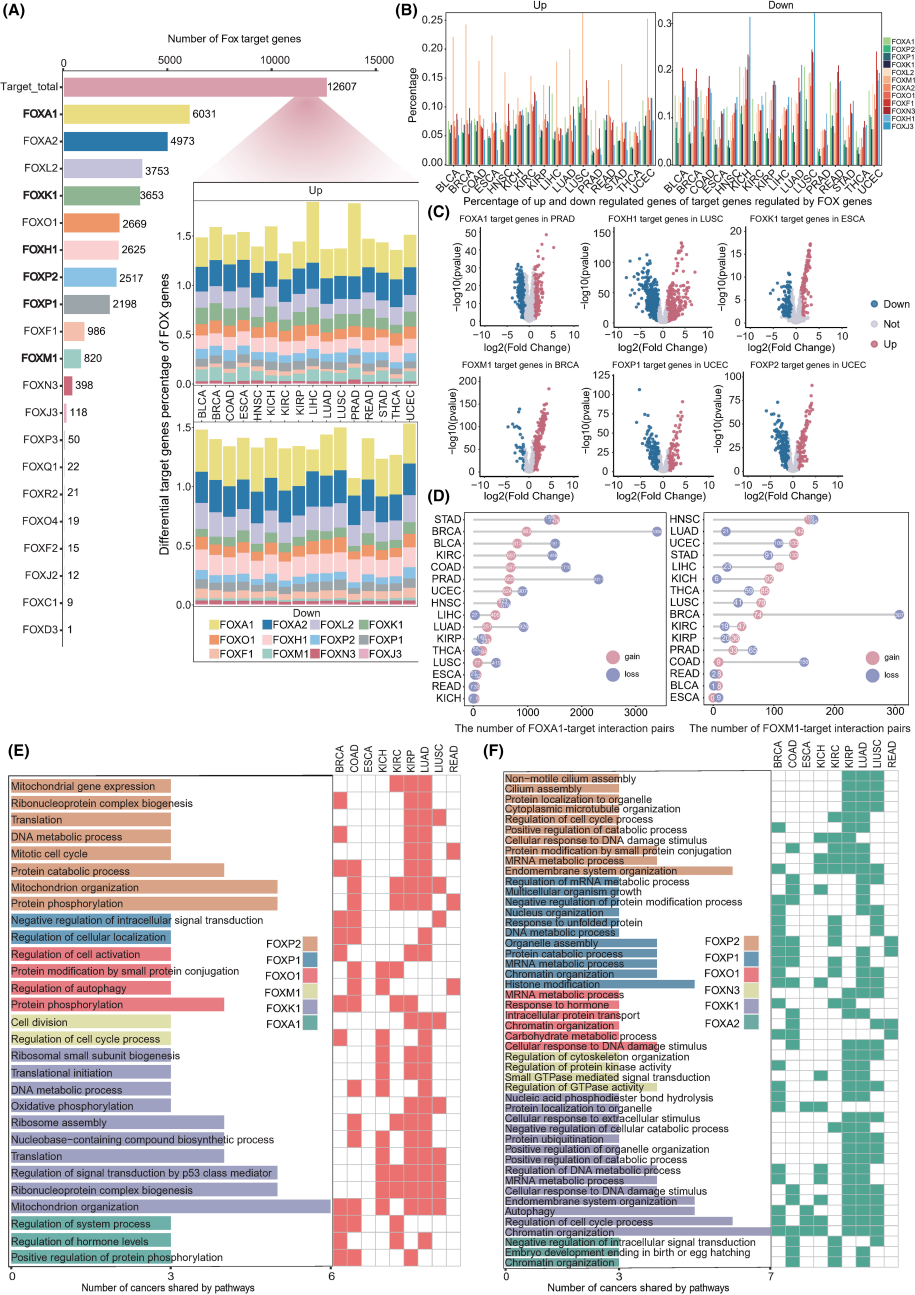
Regulatory patterns of FOX genes on target genes in cancer. (A) A bar plot showing the number of target genes for each of the 20 FOX genes. The total number of FOX target genes is shown in red after removing duplicates. The pop‐up panel showed the differential target gene (upregulated and downregulated) proportion of FOX genes. (B) The ratio of 12 FOX genes' upregulated and downregulated targets in 16 cancers, each of which has more than 100 target genes. (C) Volcano map of differential target genes of six specific FOX genes. Red denotes upregulation, blue denotes downregulation, and only significant dots with adj. P < 0.05 were shown. (D) The distribution of gain and loss FOXA1‐target and FOXM1‐target interaction pairs across cancers are shown, with numbers in the circles. Biological processes enriched by the target genes in gain (E) or loss (F) FOX‐target interaction pairs in 9 cancers, with significant changes in either cancer genome or transcriptome. The number of cancers shared by the pathways is indicated by the length of the bar, which correlates to the heatmap on the right.

Given that the FOX genes are members of an essential family of transcription factors, the target genes of FOX genes can be made to participate in biological processes by having their transcription regulated in a specific manner. We first collected 12,607 nonredundant targets of FOX genes from the ChIP‐Atlas database, with FOXA1 having the most targets (*n* = 6031) and FOXD3 having the fewest targets (*n* = 1) (Figure 4A and Figure S6). Differential gene expression analysis revealed that the distribution of genes targeted by 12 FOX genes, which have more than 100 target genes, was generally constant among cancers. It is noteworthy that despite fewer FOXM1 targets have decreased gene expression, more FOXM1 targets have increased gene expression across all 16 cancer types, which is consistent with the enhanced overall level of FOXM1 expression (Figure 4A,B). These results suggest that FOXM1 may play a crucial role in the development of cancer by regulating the expression of both its own and its target genes. Figure 4C shows the targets of six FOX genes that are dysregulated in the genome or transcriptome in particular cancers.

To eliminate the potential effects from sample size, we randomly perturbed the samples 1000 times when assessing the correlation between FOX genes and their respective target genes. We defined gain and loss FOX‐target interaction pairs to describe the changes of the correlation between FOX genes and their targets in cancer compared to normal. It is worth noting that BRCA had the most FOXA1‐ and FOXM1‐target interaction pairs, with loss pairs predominating (FDR <0.05). These results suggest that breast cancer may be more likely to develop if FOXA1 or FOXM1 target interaction is lost (Figure 4D).

We next focused on the targets of 10 key FOX genes (FOXA1, etc.) in 9 cancers with notable alterations in either cancer genome or transcriptome to gain insight into the function role of FOX targets. The shared pathways enriched by the significant targets in the gain and loss FOX‐target interaction pairs in at least three cancers were selected for further investigation, respectively. Notably, mitochondrion organization were enhanced in six out of nine cancers, pointing to the crucial function that mitochondria play in supporting cancer cell proliferation through metabolic processes (Figure 4E). Several pathways known to be involved in carcinogenesis, including cell division and regulation of cell cycle progress were enriched. In addition to analysis of functions that were enhanced, we investigated the cellular pathways that were enriched by the targets losing control by FOX genes (Figure 4F). The dysregulation of numerous fundamental biological pathways emphasises the importance of FOX gene targets in tumor development.

### 3.5 Survival prediction based on the FOX gene expression

**FIGURE 5:**
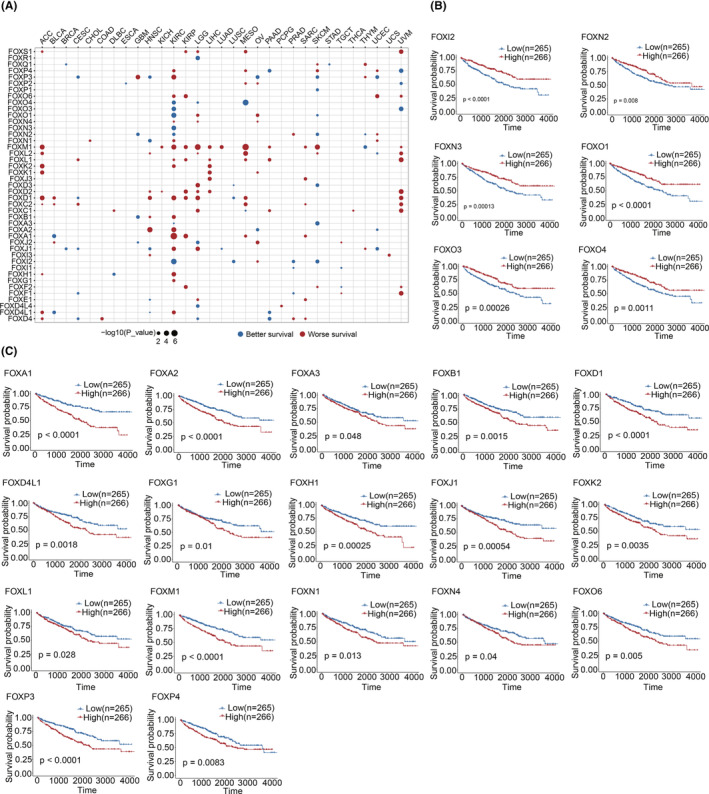
FOX gene expression as a prognostic factor for survival in multiple cancers. (A) Effect of FOX gene expression on patient survival according to Cox regression analysis. Only significant dots with a *p* < 0.05 were shown. Blue denotes better survival, whereas red denotes worse survival. Kaplan–Meier survival curves showed that high FOX gene expression was related to both improved (B) and decreased (C) overall survival. Statistical significance was assessed by log‐rank test.

Given the significant correlation between FOX genes and cancers described above, we conducted a Cox regression analysis based on FOX gene expression and clinical survival data for each cancer type to determine whether FOX gene expression could predict survival. FOX genes were found to be significantly associated with OS in 31 out of 33 cancer types (Figure 5A). Twelve tumors have a high likelihood to have FOXM1 as a risk factor, including three types of kidney malignancies, and a protective factor in one cancer (THYM). It is worth noting that approximately half (23 out of 49) of the FOX genes, including 6 protective genes (Figure 5B) and 17 risk genes (Figure 5C), were significantly associated with KIRC survival, indicating that dysregulated FOX gene expression may be crucial for KIRC survival. Validation was carried out with 101 clear cell renal cell carcinoma patients. Despite the results showing that fewer FOX genes are significantly associated with survival from kidney cancer, a similar pattern was observed (Figure S7). Taken together, these results revealed that the expression of FOX genes may have potential predictive value for survival and possible clinical implications in certain cancer contexts.

### 3.6 Therapeutic potential of FOX genes in cancer

**FIGURE 6:**
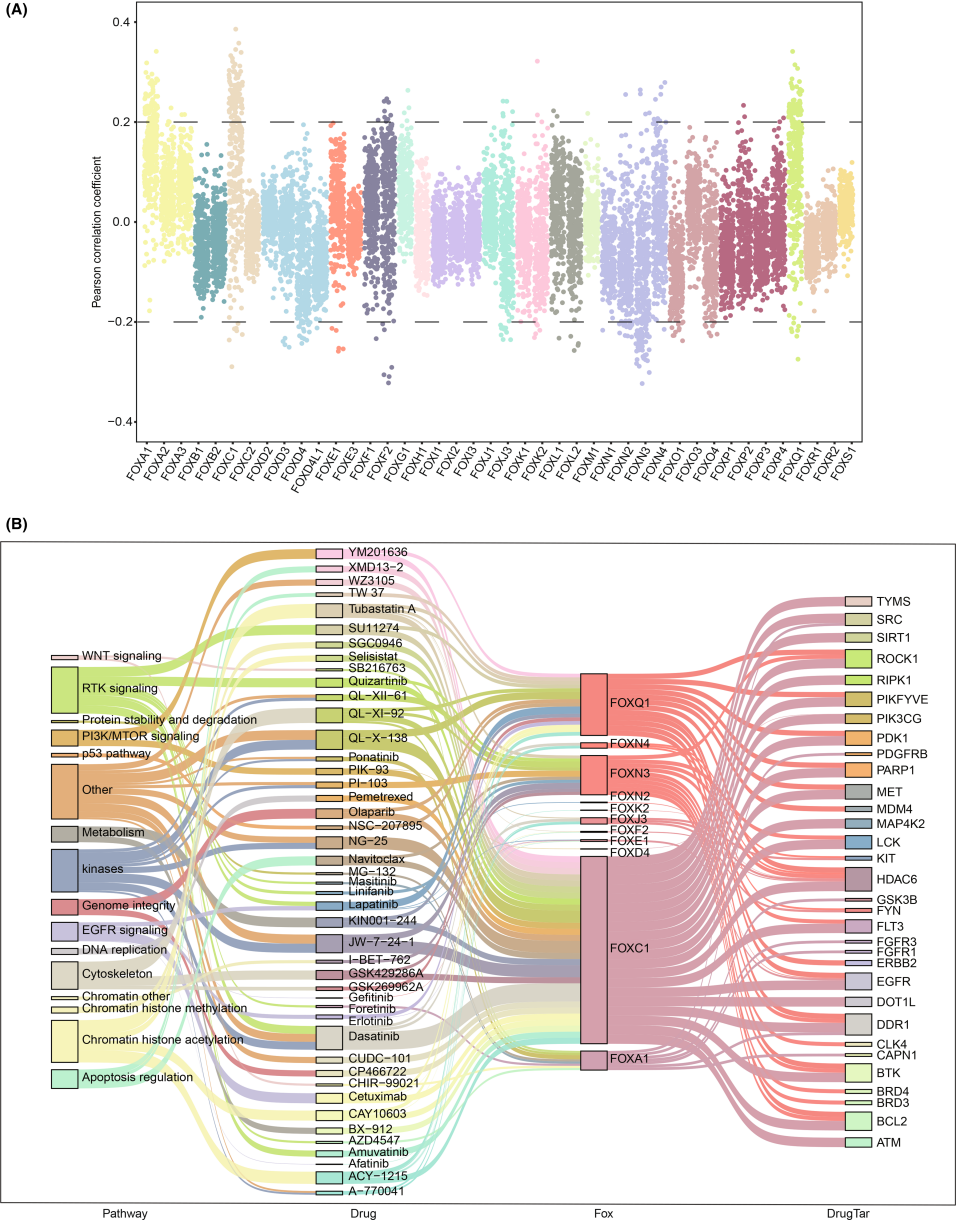
Potential drug targets of FOX genes in cancer. (A) The Pearson correlation coefficients between FOX gene expression and drug sensitivity (IC50). (B) Sankey diagram illustrating the relationships between FOX genes, drugs, drug targeted genes, and associated cancer signaling pathways.

In addition to examining the prognostic value of FOX genes in survival, we also investigated the role that FOX genes play in drug implementations. A total of 457 FOX gene‐drug correlations were successfully identified, involving 26 FOX genes and 184 drugs, using drug sensitivity and gene expression data from the cell‐lines database Genomics of Drug Sensitivity in Cancer (GDSC) (Figure 6A and Table S6). To better understand the therapeutic role of FOX genes, we further included the information of cancer signaling pathways and drug targeted genes. As a result, 103 significant correlations between 11 FOX genes, including FOXA1, FOXN3, and FOXQ1, and 32 drug targets like FGFR1, HDAC6, and EGFR were found (Figure 6B and Table S7). Some of our results, such as the EGFR signaling pathway‐erlotinib‐FOXQ1‐EGFR axis, is consistent with the previous studies showing that FOXQ1 promotes the development of angiogenic mimicry, which accelerates the spread of nasopharyngeal carcinoma, and that EGFR inhibitors can effectively suppress this process.^35^ These results validated the critical functions of FOX genes in the genesis of cancer and their potential therapeutic value.

### 3.7 FOX2Cancer: a web‐based resource for FOX gene family in human cancers

**FIGURE 7:**
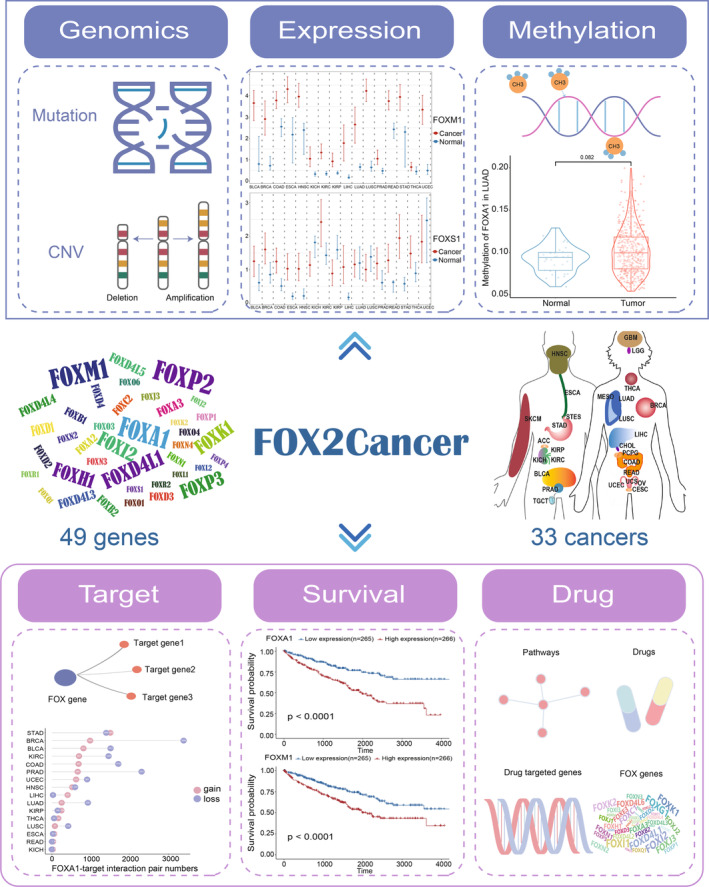
Overview of FOX2Cancer with six functional modules.

To help researchers better understand and thoroughly query the spectrum of multi‐omics molecular features of the FOX gene family, we developed the FOX2Cancer database (Figure 7), which is freely accessible at http://hainmu‐biobigdata.com/FOX2Cancer.FOX2Cancer allows users to conduct a multi‐omics study for certain cancer types and obtain the results by searching for the FOX genes of interest, which can be categorized into the following six function modules: (i) in genomics analysis module, users can query the results of genomics events of FOX genes, including somatic mutation spectrum and copy number alterations. (ii) In expression analysis module, users can either query the level of expression in a particular cancer or plot the expression profiles of any FOX gene across 16 cancers. (iii) In methylation analysis module, users can search for the DNA methylation level of the FOX genes in related cancers. (iv) In target gene module, users can research details about the FOX gene targets, including their expression levels in tumors and the relationships between FOX genes and their targets. (v) In survival analysis module, for each cancer type, users can perform analysis and generate survival curves based on FOX gene expression level. (vi) In drug analysis module, users can investigate the FOX genes' possible therapeutic targets and related drugs. Users can download all of the FOX2Cancer data for their own customized research. Overall, FOX2Cancer will provide a comprehensive and readily accessible webserver to investigate the multi‐omics molecular characteristics of FOX gene family across 33 human cancer types.

## 4 DISCUSSION

Our study reveals the full omics picture of the FOX gene family and its clinical implications, including survival prediction value and possible therapeutic targets, by conducting an integrated analysis covering multi‐omics and clinical data of the FOX gene family from over 11,000 pan‐cancer patients. In agreement with previous reports in a single FOX gene,^18^, ^19^ heterogeneity of FOX gene expression across cancer types was found in our results, and it was partially contributed by genomic or epigenomic changes. Additionally, we discovered previously unreported heterogeneous expression pattern and skewed cancer‐type genomic alteration pattern among the FOX gene family members. Moreover, FOX gene co‐expression analysis demonstrate that FOX genes exert their functions by controlling the expression of both their own and target genes. All the results have been integrated into the first FOX gene knowledgebase, FOX2Cancer, for individualized use by researchers and clinicians. These findings provide a multi‐omics overview of the molecular landscape of the FOX gene family in pan‐cancer and highlight the significance of FOX genes as potential clinical targets for cancer research.

The expression pattern of human FOX transcription factors has been investigated in normal tissues.^1^, ^12^ In general, both GTEx expression data^36^ and Unigene EST profiles demonstrate that the FOX gene family exhibits two main expression patterns: nearly ubiquitous expression in all tissues (~ 25% of FOX genes) and selective expression to specific tissue(s) (~75% of FOX genes). The entire FOX gene family's expression pattern across various cancers has never been reported prior to a recent study by Yin et al.^37^ Due to the study's main findings concentrating on other areas, however, few conclusive findings were discussed on this. Here we provide the first expression landscape of FOX gene family in 16 major cancer types from The Cancer Genome Atlas (TCGA). More than 80% of FOX genes have differential expression in at least one type of cancer (log2‐fold change > or < −1 and adjusted *p* < 0.05), which suggests that FOX genes are generally dysregulated in tumor development with a more complicated expression pattern. In agreements with previous reports, FOXM1 gene expression is elevated in all cancer types and correlates with its copy number amplification,^18^, ^38^ and FOXS1 were upregulated in most cancers but downregulated in KIRP,^19^ indirectly validated the accuracy of our analysis. It is worth noting that from a multi‐omics perspective, we first provide the overall picture of the FOX gene family and the intricate connections between transcriptomic changes, genomic modifications, and epigenomic abnormalities of the FOX gene family. However, we were unable to dissect underlying relationships of each of the 49 FOX genes' multi‐omics data in this paper. Additionally, even though we can now infer that the FOX co‐expression relationships are globally disrupted in cancer tissues, it is still unclear how the centric FOX regulators shifted from normal (FOXN3 and FOXJ2) to cancer (FOXL1 and FOXD4L). Thus, we have provided all omics findings in FOX2Cancer database for future research by us or other researchers.

Several of the FOX factors are indirect targets of cancer drugs and prognostic biomarkers.^39^, ^40^, ^41^ Overexpression of FOXM1, FOXC2 and FOXP1 have been shown to be important in tumor development, drug resistance, metastasis and poor prognosis in a variety of cancer types.^42^, ^43^, ^44^, ^45^, ^46^, ^47^, ^48^, ^49^ FOXM1‐regulated gene signature has been demonstrated to be a reliable diagnostic and prognostic marker to improve early squamous cell carcinoma patient drug treatment responsiveness.^50^ Recent approaches aiming to regulate transcription factor activity have been proposed, making it possible to successfully target FOX genes in cancer therapy.^51^, ^52^, ^53^, ^54^ Therapeutic effects of chemotherapy drugs like paclitaxel, imatinib, and doxorubicin are achieved by targeting FOXO3a through oncogenic kinases (AKT, IKK, and ERK) and numerous signaling pathways.^55^, ^56^ Our findings confirm that EGFR inhibitor erlotinib can suppress angiogenic mimicry by EGFR targeting of FOXQ1, and re‐emphasize its crucial significance as new therapeutic targets. From this perspective, here we predict over a hundred cancer signaling pathway‐drug‐FOX gene‐drug target axis. Further functional research and clinical trials are required to determine which FOX gene might be suitable targets in the fight against cancer.

FOX2Cancer webserver provided in this study serve as a time‐saving and effective resource to access preliminary multi‐omics results of any FOX gene of interest, allowing researchers to perform fast hypothesis testing. Users can generate bioinformatics analysis on FOX genes, such as differential gene expression and survival analysis, without any programming experience, and all of the processed data from this study is available for download. In conclusion, this study expands our understanding of the molecular characteristics of FOX genes in carcinogenesis and may aid in the future development of FOX‐targeting therapies.

## AUTHOR CONTRIBUTIONS

**Xiaoman Bi:** Conceptualization (equal); formal analysis (equal); funding acquisition (supporting); supervision (equal); writing – original draft (lead); writing – review and editing (lead). **Dehua Zheng:** Data curation (lead); formal analysis (lead); methodology (lead); writing – original draft (equal); writing – review and editing (equal). **Jiale Cai:** Methodology (equal); resources (lead); software (lead); visualization (equal); writing – original draft (supporting). **Dahua Xu:** Data curation (equal); formal analysis (equal); methodology (equal); software (equal). **Liyang Chen:** Formal analysis (supporting). **Zhizhou Xu:** Formal analysis (supporting). **Meng Cao:** Formal analysis (supporting). **Peihu Li:** Software (supporting). **Yutong Shen:** Software (supporting). **Hong Wang:** Project administration (supporting); supervision (supporting). **Wuping Zheng:** Project administration (supporting); supervision (supporting). **Deng Wu:** Software (lead). **Shaojiang Zheng:** Conceptualization (lead); funding acquisition (lead); supervision (equal); writing – review and editing (equal). **Kongning Li:** Conceptualization (lead); funding acquisition (lead); project administration (lead); supervision (lead); writing – review and editing (equal).

## FUNDING INFORMATION

This work was supported by Hainan Province Science and Technology special (ZDYF2021SHFZ097, ZDYF2021SHFZ248, ZDYF2020132 and ZDYF2022SHFZ065); Hainan Provincial Natural Science Foundation of China (822QN462 and 823RC581); The National Natural Science Foundation of China (32160152 and 81960528); Major Science and Technology Program of Hainan Province (ZDKJ2021040); Postdoctoral Science Foundation of Hainan Province; Youth Science and Technology Talent Innovation Program of Hainan Association for Science and Technology (QCQTXM202212); Bioinformatics for Major Diseases Science Innovation Group of Hainan Medical University; the specific research fund of The Innovation Platform for Academicians of Hainan Province (YSPTZX202208) and Hainan Province Clinical Medical Center (QWYH2021276).

## CONFLICT OF INTEREST STATEMENT

The authors declare that they have no conflict of interest related to this work.

## Supporting information


Figure S1.

Table S2.

## Data Availability

The multi‐omic and clinical data can be found at UCSC Xena (http://xena.ucsc.edu/). Software and resources used for the analyses are described in each method section.
