# Supplementary material for: Pan‐cancer analyses reveal multi‐omic signatures and clinical implementations of the forkhead‐box gene family
Source: Cancer Med. 2023 Jul 4;12(16):17428–44. doi: 10.1002/cam4.6312 (PMC10501247; doi:10.1002/cam4.6312)
Supplement: Supplementary file 1 — Figure S1. [file CAM4-12-17428-s002.docx]

Supplementary Information for:

Pan-cancer analyses reveal multi-omic signatures and clinical implementations of the forkhead-box gene family

Bi et al.


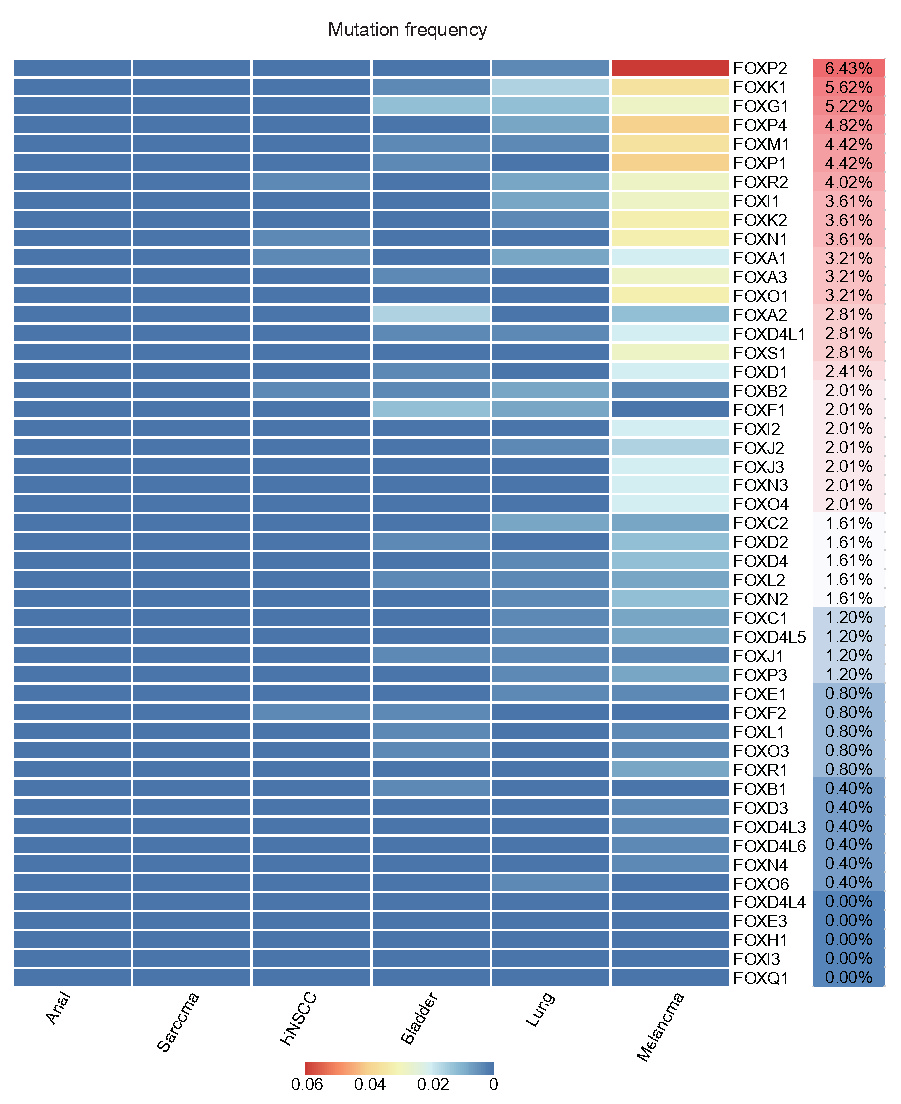


**Supplementary Fig.1** Heatmap showing the frequency of somatic FOX gene mutations across six cancers.


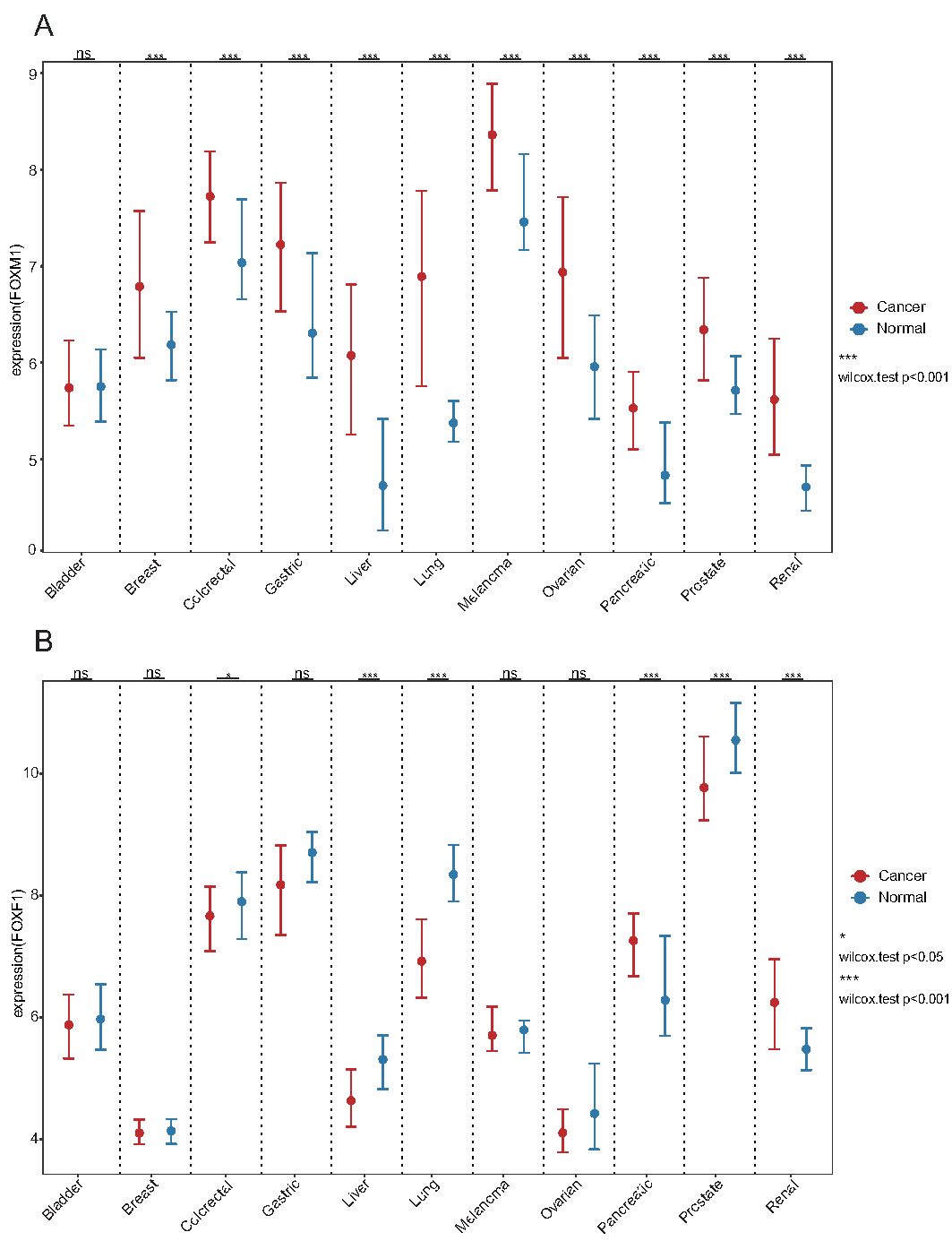


**Supplementary Fig.2**. The expression of FOXM1 (A) and FOXF1 (B) in cancer and normal samples across cancer types was shown using box plots. The middle line represents the median, and the upper and lower quartiles are the box’s boundaries. ns, not significant.


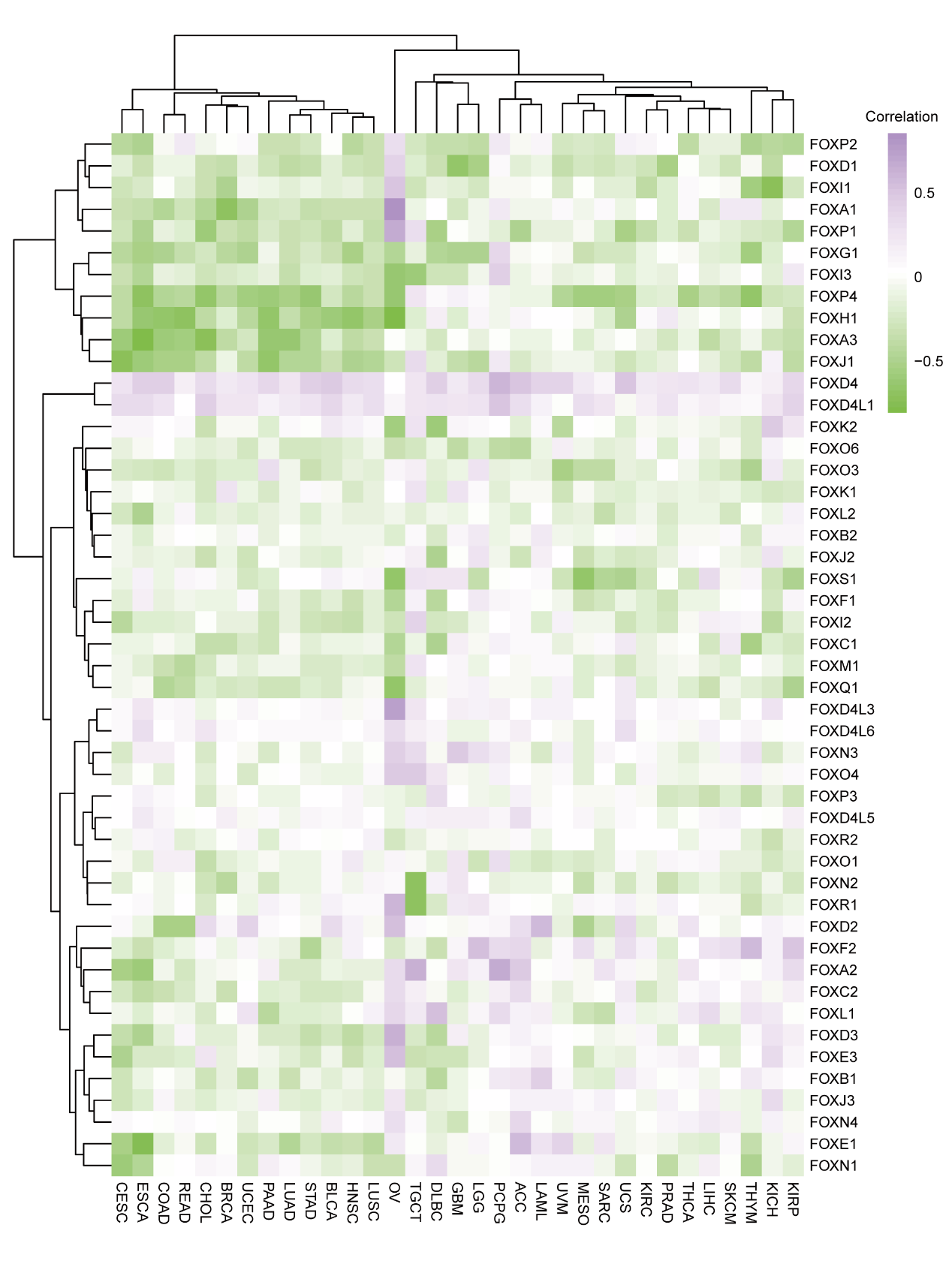


**Supplementary Fig.3** Heat map of correlation between FOX genes methylation level and expression.

**
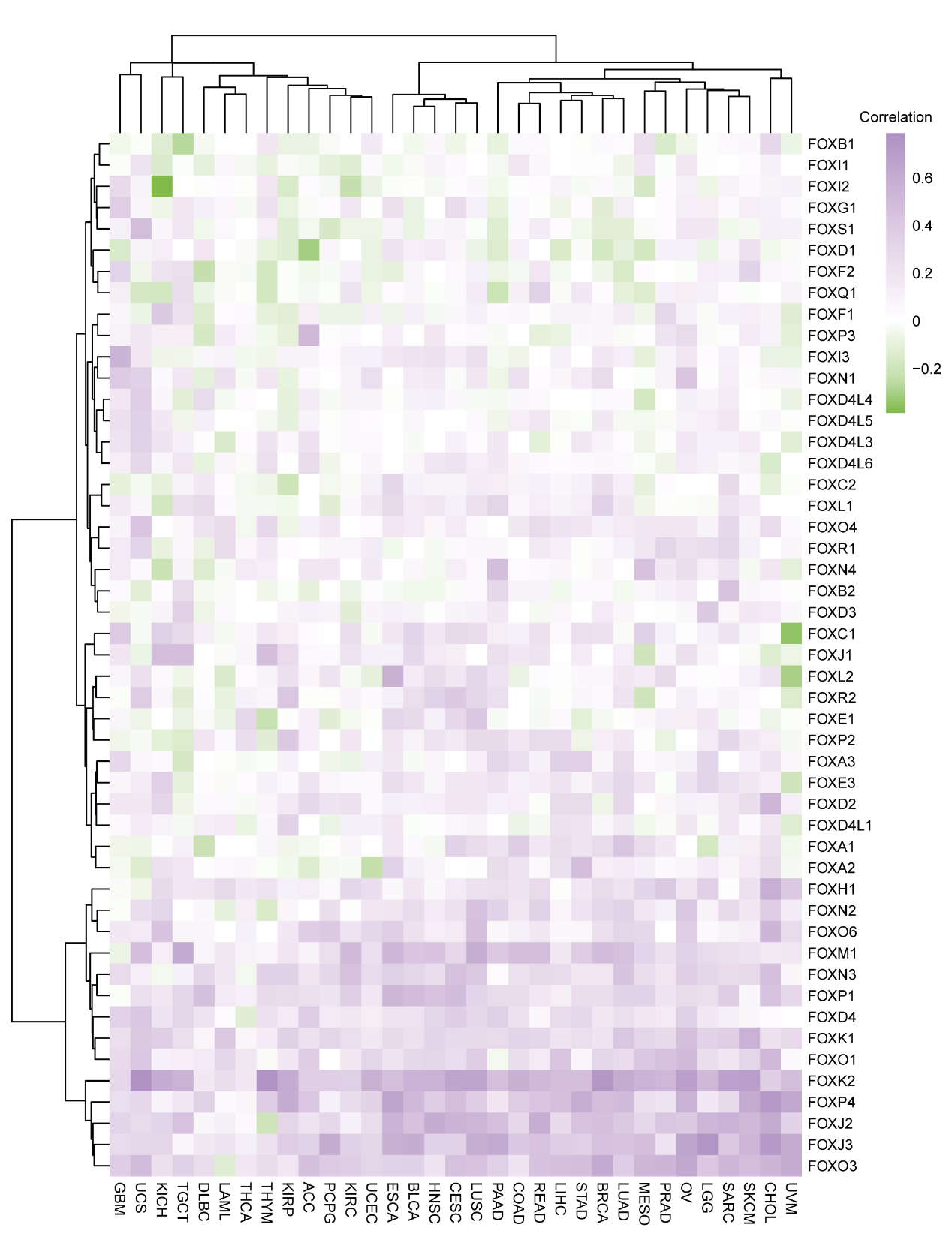
**

**Supplementary Fig.4** Heat map of correlation between FOX genes CNV alteration level and expression.


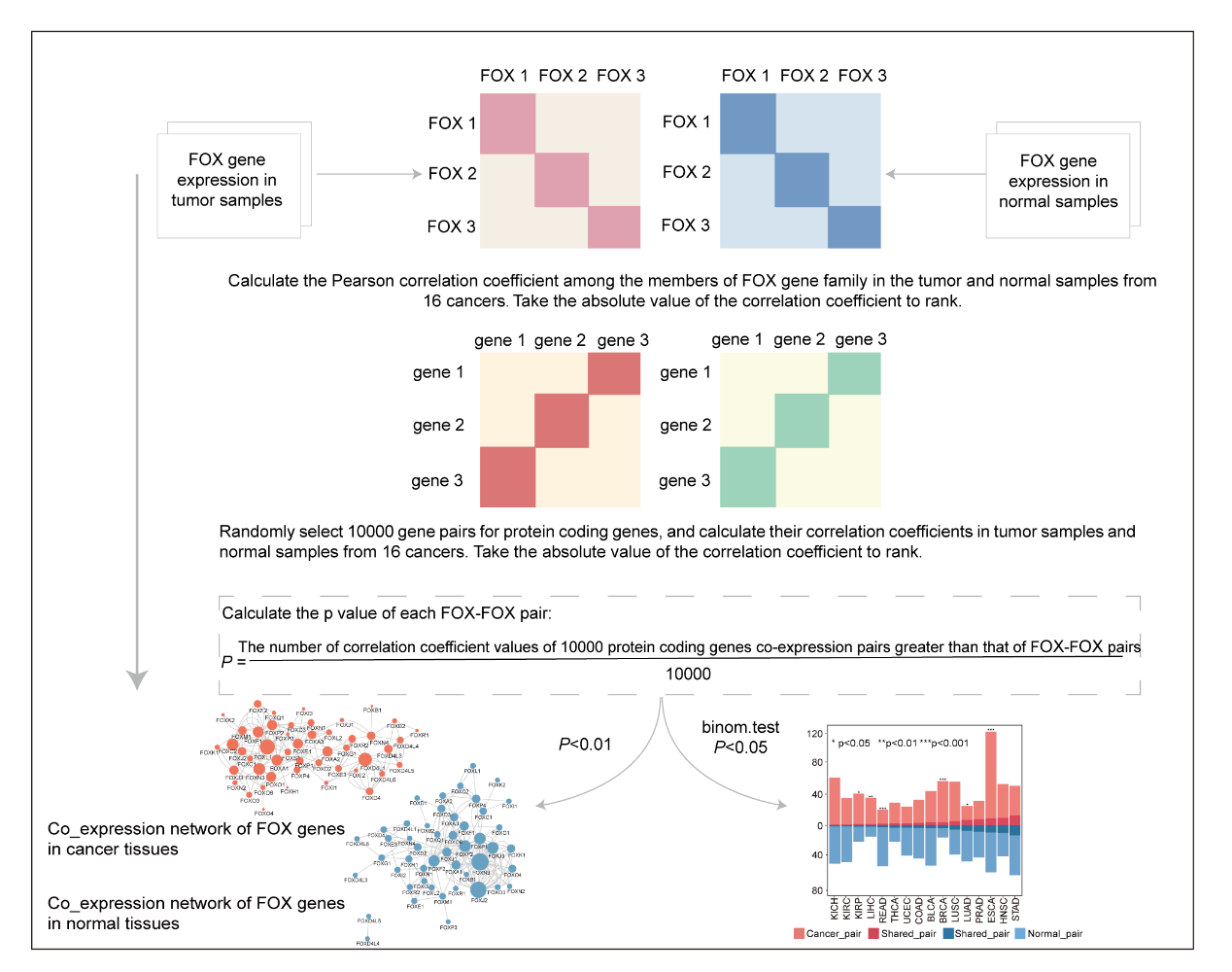


**Supplementary Fig.5** Flow of co-expression analysis of FOX gene family.


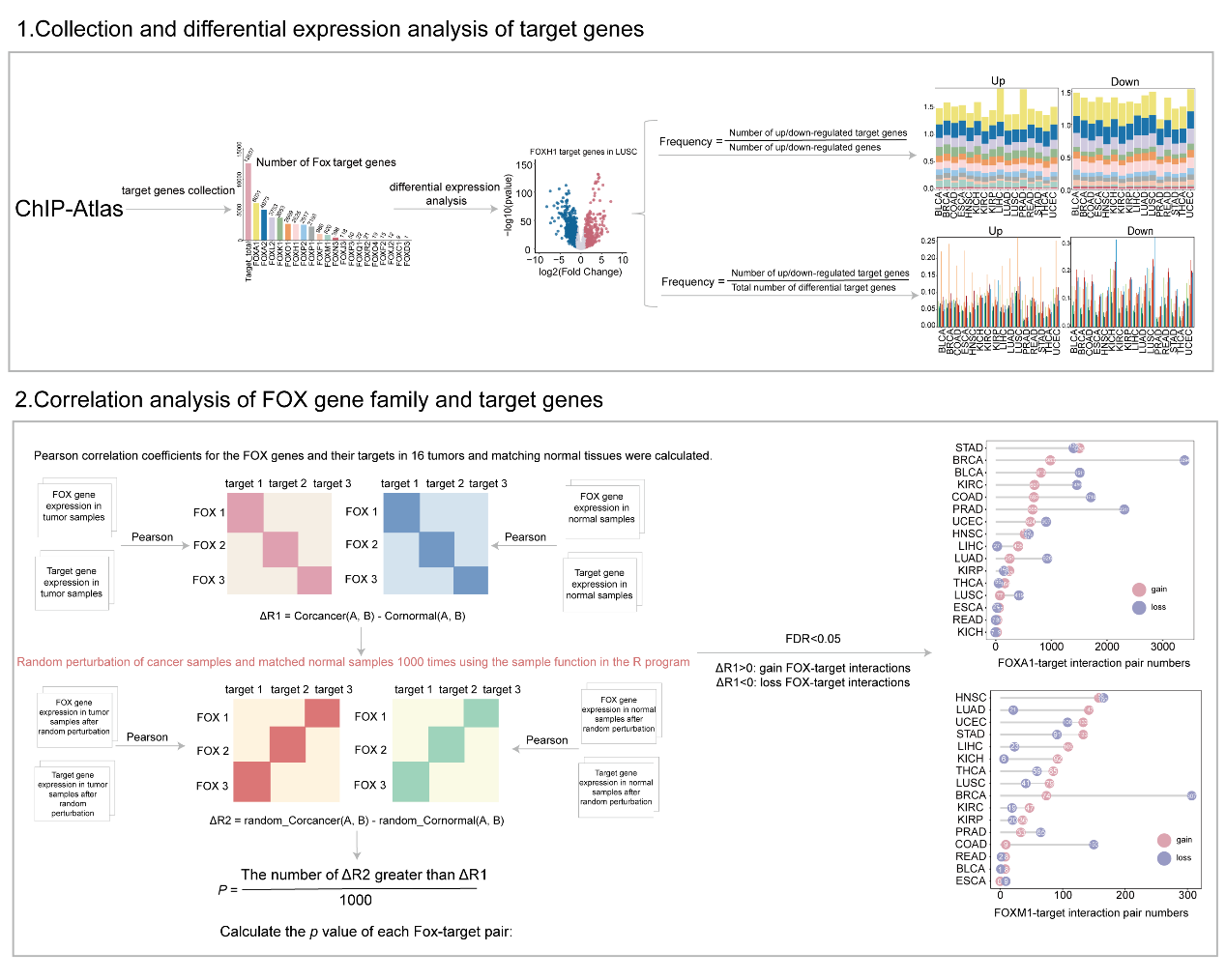


**Supplementary Fig.6** Analysis process of FOX genes and target genes.


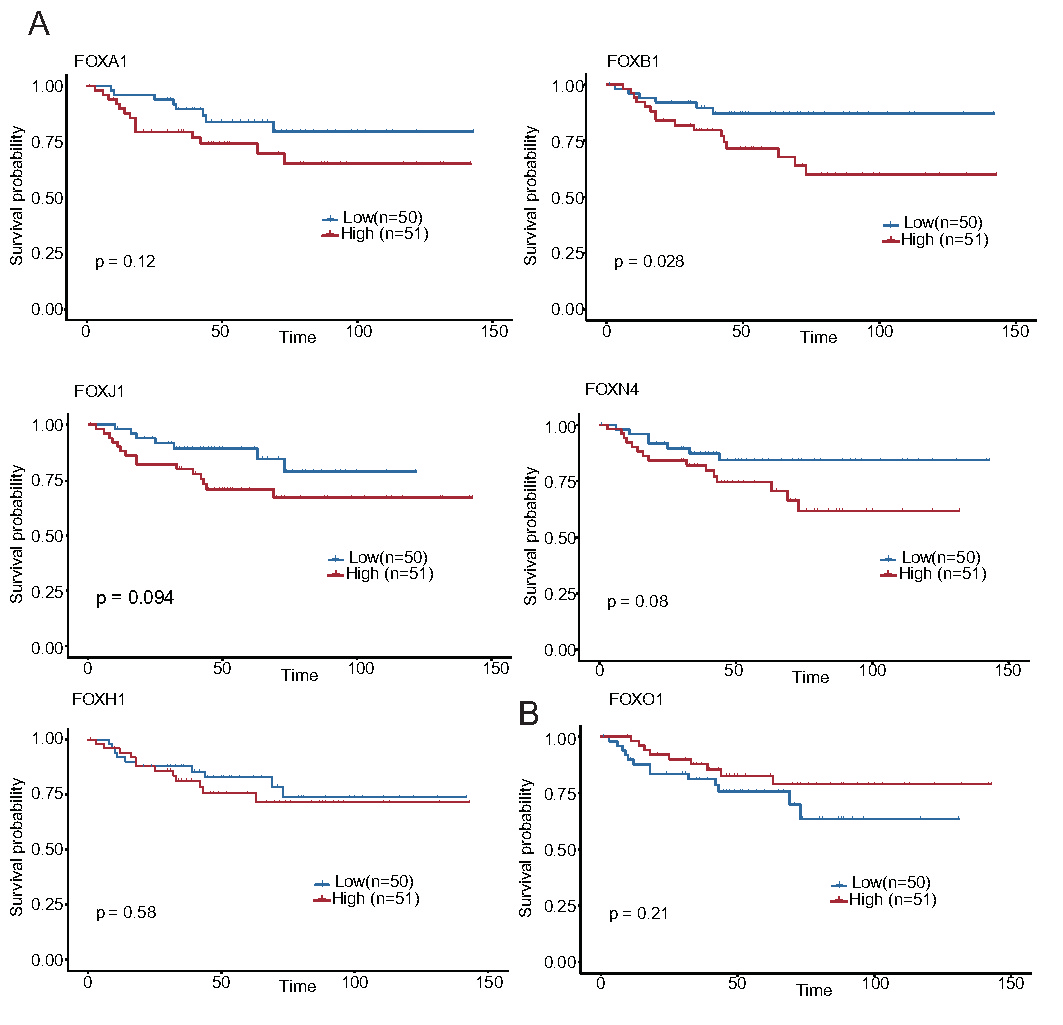


**Supplementary Fig.7** Kaplan–Meier survival curves showed that high FOX gene expression was related to both improved (A) and decreased (B) overall survival. Statistical significance was assessed by log-rank test.
